# Supplementary material for: Identification of Key Genes during Ethylene-Induced Adventitious Root Development in Cucumber (Cucumis sativus L.)
Source: Int J Mol Sci. 2022 Oct 26;23(21):12981. doi: 10.3390/ijms232112981 (PMC9658848; doi:10.3390/ijms232112981)
Supplement: Supplementary file 1 [file ijms-23-12981-s001.zip › ijms-1968823-supplementary/Supplementary Table S10.pdf]

Supplementary Table S10. Expression patterns of DEGs related to plant hormone transduction during adventitious root development in cucumber.

Gene id	Gene name	FPKM(the control)	FPKM(ETH)	log2FC	Gene description	Up/down
101205750	*LOC101205750*	413.0915296	201.3487135	-1.03415524	auxin transporter-like protein 5	down
101206566	*CS-AUX1*	436.7514737	284.2922061	-0.617977602	auxin transporter-like protein 4	down
101218948	*LOC101218948*	2093.509986	1224.462628	-0.774066066	auxin-induced protein AUX28-like	down
101209970	*LOC101209970*	3667.783946	4781.92876	0.382675861	auxin-responsive protein IAA26 isoform X2	up
101215306	*LOC101215306*	386.9786552	269.3011553	-0.520486582	auxin-responsive protein IAA13 isoform X1	down
101223028	*LOC101223028*	240.5546524	126.602255	-0.929375785	auxin-responsive protein IAA4	down
101206247	*LOC101206247*	205.9568512	96.16620169	-1.097855929	indole-3-acetic acid-amido synthetase GH3.17	down
101217643	*LOC101217643*	8.494299009	0.600228117	-3.724370843	auxin-responsive protein SAUR50	down
101209777	*LOC101209777*	497.6096131	281.3851537	-0.822251144	histidine kinase 4 isoform X1	down
101213450	*LOC101213450*	231.6813887	117.8538294	-0.974345792	DELLA protein GAI	down
101205993	*LOC101205993*	4054.074448	5026.203158	0.310178999	transcription factor PIF5	up
101208823	*LOC101208823*	2189.685268	2963.970499	0.436698838	abscisic acid receptor PYL8	up
101204882	*LOC101204882*	704.3018817	992.5458647	0.495171389	abscisic acid receptor PYR1	up
101210262	*LOC101210262*	4528.635526	5983.85308	0.401843236	abscisic acid receptor PYL5	up
101209540	*LOC101209540*	2104.752403	2871.807768	0.448377598	serine/threonine-protein kinase SRK2G isoform X1	up
101214088	*LOC101214088*	1827.744737	2264.624188	0.309041459	serine/threonine-protein kinase SRK2H	up
101222720	*LOC101222720*	701.881049	962.5480622	0.455126028	ABSCISIC ACID-INSENSITIVE 5-like protein 2	up
101205786	*CS-ERS*	2058.433654	1014.125839	-1.02145856	ethylene response sensor 1	down

101207154	*LOC101207154*	588.9532738	422.8393976	-0.527090865	EIN3-binding F-box protein 1-like	down
101206564	*LOC101206564*	159.0363113	65.46705306	-1.280037711	ethylene-response factor C3	down
101220325	*LOC101220325*	320.9817361	132.8811344	-1.271089931	ethylene-response factor C3	down
101216386	*LOC101216386*	725.1006672	1588.333778	1.13084843	BRASSINOSTEROID INSENSITIVE 1-associated receptor kinase 1	up
101217842	*XTH3*	1321.364173	2420.072446	0.87298424	probable xyloglucan endotransglucosylase/hydrolase protein 23-like precursor	up
101212037	*LOC101212037*	1793.913492	2334.948208	0.380502206	jasmonoyl--L-amino acid synthetase JAR6	up
101210308	*LOC101210308*	46.7542977	88.70375995	0.926697125	transcription factor TGA2.3 isoform X2	up
116401613	*LOC116401613*	36.23236296	174.2655857	2.268223637	transcription factor TGA1-like isoform X1	up
