# Supplementary material for: Identification of Key Genes during Ethylene-Induced Adventitious Root Development in Cucumber (Cucumis sativus L.)
Source: Int J Mol Sci. 2022 Oct 26;23(21):12981. doi: 10.3390/ijms232112981 (PMC9658848; doi:10.3390/ijms232112981)
Supplement: Supplementary file 1 [file ijms-23-12981-s001.zip › ijms-1968823-supplementary/Supplementary Table S5.pdf]

Supplementary Table S5. Expression patterns of DEGs related to starch and sucrose metabolism during adventitious root development in cucumber.

**Gene id**	**Gene name**	**FPKM(the control)**	**FPKM(ETH)**	**log2FC**	**Gene description**	**Up/down**
101216058	*LOC101216058*	211.5413126	119.425566	-0.824957455	hexokinase-2, chloroplastic	down
101214838	*LOC101214838*	260.6648309	137.1208053	-0.929520598	beta-glucosidase 11-like	down
101204921	*LOC101204921*	1208.417721	604.7844765	-0.997196723	endoglucanase 17	down
101203271	*LOC101203271*	2681.878408	1057.937086	-1.342280383	endoglucanase 6	down
101222401	*LOC101222401*	156.0698503	54.10416324	-1.52867718	endoglucanase 9	down
101221022	*LOC101221022*	2034.689121	1483.251714	-0.455433558	probable sucrose-phosphate synthase 1	down
101211097	*LOC101211097*	287.9351312	152.5013089	-0.913794203	probable trehalose-phosphate phosphatase J	down
101212172	*LOC101212172*	287.9351312	152.5013089	-0.569243815	trehalose-phosphate phosphatase A isoform X1	down
101211461	*LOC101211461*	14140.57061	7504.866151	-0.913911787	sucrose synthase 5-like	down
101221739	*LOC101221739*	5055.58948	3130.246729	-0.691752461	beta-amylase 1, chloroplastic	down
101213455	*LOC101213455*	1254.953454	731.1416041	-0.779306025	beta-amylase 3, chloroplastic	down
101222005	*LOC101222005*	702.1943017	967.2566244	0.462459969	beta-amylase isoform X1	up
101215687	*LOC101215687*	16778.54928	26114.06217	0.638245864	1,4-alpha-glucan-branching enzyme 1, chloroplastic/amyloplastic	up
101221562	*LOC101221562*	833.7599588	579.6210721	-0.524554892	glucose-1-phosphate adenylyltransferase large subunit 1	down
101208904	*LOC101208904*	1744.897462	1046.879964	-0.736968108	glucose-1-phosphate adenylyltransferase large subunit 1	down
101208605	*LOC101208605*	2614.131422	1927.173602	-0.439326203	glucose-1-phosphate adenylyltransferase large subunit 3, chloroplastic/amyloplastic	down
101209348	*LOC101209348*	424.2250124	302.0410415	-0.489680863	isoamylase 3, chloroplastic isoform X1	down
