# Supplementary material for: Identification of Key Genes during Ethylene-Induced Adventitious Root Development in Cucumber (Cucumis sativus L.)
Source: Int J Mol Sci. 2022 Oct 26;23(21):12981. doi: 10.3390/ijms232112981 (PMC9658848; doi:10.3390/ijms232112981)
Supplement: Supplementary file 1 [file ijms-23-12981-s001.zip › ijms-1968823-supplementary/Supplementary Table S6.pdf]

Supplementary Table S6. Expression patterns of DEGs related to glycolysis / gluconeogenesis, TCA cycle and oxidative phosphorylation during adventitious root development in cucumber.

Gene id	Gene name	FPKM(the control)	FPKM(ETH)	log2FC	Gene description	Up/down
101216058	*LOC101216058*	211.5413126	119.425566	-0.824957455	hexokinase-2, chloroplastic	down
101202739	*LOC101202739*	519.2778174	380.5966674	-0.447867239	ATP-dependent 6-phosphofructokinase 7	down
101215219	*LOC101215219*	89.86761101	30.10202069	-1.574815215	pyrophosphate--fructose 6-phosphate 1-phosphotransferase subunit beta	down
101219631	*LOC101219631*	893.4465961	1162.772511	0.379958475	phosphoglycerate kinase, cytosolic isoform X1	up
101215513	*LOC101215513*	4051.997044	3189.331605	-0.345317487	phosphoglycerate kinase, cytosolic	down
101206620	*LOC101206620*	473.5240406	307.2283788	-0.624220059	pyruvate kinase 2, cytosolic	down
101220786	*LOC101220786*	413.6381879	280.1318568	-0.56075632	plastidial pyruvate kinase 1, chloroplastic	down
101215151	*LOC101215151*	661.7114477	504.9215729	-0.389212571	dihydrolipoyllysine-residue acetyltransferase component 5 of pyruvate dehydrogenase complex, chloroplastic	down
101220730	*LOC101220730*	547.9298619	383.0058965	-0.516365518	dihydrolipoyllysine-residue acetyltransferase component 4 of pyruvate dehydrogenase complex, chloroplastic	down
101204247	*LOC101204247*	1273.343042	891.5546015	-0.513627027	pyruvate dehydrogenase E1 component subunit beta-3, chloroplastic	down
101219569	*LOC101219569*	375.6855858	500.8700405	0.415714789	aldehyde dehydrogenase family 3 member H1	up
101211996	*LOC101211996*	2260.624756	1643.027867	-0.459858207	alcohol dehydrogenase-like 7	down
101215151	*LOC101215151*	661.7114477	504.9215729	-0.389212571	dihydrolipoyllysine-residue acetyltransferase component 5 of pyruvate	down

					dehydrogenase complex, chloroplastic	
101220730	*LOC101220730*	547.9298619	383.0058965	-0.516365518	dihydrolipoyllysine-residue acetyltransferase component 4 of pyruvate dehydrogenase complex, chloroplastic	down
101204247	*LOC101204247*	1273.343042	891.5546015	-0.513627027	pyruvate dehydrogenase E1 component subunit beta-3, chloroplastic	down
101209539	*LOC101209539*	250.3699517	121.3426549	-1.042566389	ATP-citrate synthase beta chain protein 2	down
101202770	*LOC101202770*	370.5677261	240.9848612	-0.621522706	isocitrate dehydrogenase [NADP]	down
101208633	*LOC101208633*	1809.730389	1463.980823	0.30559677	NADH dehydrogenase [ubiquinone] 1 alpha subcomplex subunit 2	up
11123919	*nad5*	52.10098238	19.30980126	1.431974932	nad5; NADH dehydrogenase subunit 5	up
101205555	*LOC101205555*	39.46192654	93.58787469	-1.23829297	uncharacterized protein LOC101205555	down
101213505	*LOC101213505*	6719.296802	4493.616087	0.580362	plasma membrane ATPase 4	up
