# Supplementary material for: Identification of Key Genes during Ethylene-Induced Adventitious Root Development in Cucumber (Cucumis sativus L.)
Source: Int J Mol Sci. 2022 Oct 26;23(21):12981. doi: 10.3390/ijms232112981 (PMC9658848; doi:10.3390/ijms232112981)
Supplement: Supplementary file 1 [file ijms-23-12981-s001.zip › ijms-1968823-supplementary/Supplementary Table S7.pdf]

Supplementary Table S7. Expression patterns of DEGs related to fatty acid biosynthesis and fatty acid degradation during adventitious root development in cucumber.

Gene id	Gene name	FPKM(the control)	FPKM(ETH)	log2FC	Gene description	Up/down
101221009	*LOC101221009*	375.7713542	521.0006339	0.471662802	NADPH-dependent aldehyde reductase-like protein, chloroplastic	up
101210955	*LOC101210955*	562.2575873	375.8738699	-0.58118772	palmitoyl-acyl carrier protein thioesterase, chloroplastic	down
101207461	*LOC101207461*	8358.496775	12339.03771	0.561967871	long chain acyl-CoA synthetase 6, peroxisomal	up
101212158	*LOC101212158*	762.3602457	1062.390833	0.479399183	long chain acyl-CoA synthetase 8 isoform X1	up
101207461	*LOC101207461*	8358.496775	12339.03771	0.561967871	long chain acyl-CoA synthetase 6, peroxisomal	up
101212158	*LOC101212158*	762.3602457	1062.390833	0.479399183	long chain acyl-CoA synthetase 8 isoform X1	up
101210295	*LOC101210295*	6764.986849	9382.391797	0.471956374	glyoxysomal fatty acid beta-oxidation multifunctional protein MFP-a	up
101217710	*LOC101217710*	7463.409009	11385.71581	0.609384671	3-ketoacyl-CoA thiolase 2, peroxisomal-like	up
101212707	*LOC101212707*	1515.957865	1852.264464	0.289143779	acetyl-CoA acetyltransferase, cytosolic 1	up
101211996	*LOC101211996*	2260.624756	1643.027867	-0.459858207	alcohol dehydrogenase-like 7	down
101219569	*LOC101219569*	375.6855858	500.8700405	0.415714789	aldehyde dehydrogenase family 3 member H1	up
101214516	*LOC101214516*	18.84412921	1.301779452	-3.821959353	cytochrome P450 86A1	down
