# Supplementary material for: Identification of Key Genes during Ethylene-Induced Adventitious Root Development in Cucumber (Cucumis sativus L.)
Source: Int J Mol Sci. 2022 Oct 26;23(21):12981. doi: 10.3390/ijms232112981 (PMC9658848; doi:10.3390/ijms232112981)
Supplement: Supplementary file 1 [file ijms-23-12981-s001.zip › ijms-1968823-supplementary/Supplementary Table S8.pdf]

Supplementary Table S8. Expression patterns of DEGs related to phenylalanine metabolism during adventitious root development in cucumber.

Gene id	Gene name	FPKM(the control)	FPKM(ETH)	log2FC	Gene description	Up/down
101218856	*LOC101218856*	10.16267166	44.9672348	-2.130183977	phenylalanine ammonia-lyase	down
101217299	*LOC101217299*	1696.854769	1104.228048	0.62006779	aspartate aminotransferase, mitochondrial	up
101210998	*LOC101210998*	1076.283911	779.1511287	0.466434702	primary amine oxidase isoform X1	up
101213059	*LOC101213059*	4122.999035	2206.139493	0.902290297	primary amine oxidase	up
101212818	*LOC101212818*	322.4630146	182.6097464	0.82271878	primary amine oxidase	up
101214819	*LOC101214819*	621.4243791	448.9154708	0.4693775	probable amidase At4g34880	up
101204792	*LOC101204792*	103.8942071	169.8267418	-0.709438096	probable amidase At4g34880	down
