# Supplementary material for: Identification of Key Genes during Ethylene-Induced Adventitious Root Development in Cucumber (Cucumis sativus L.)
Source: Int J Mol Sci. 2022 Oct 26;23(21):12981. doi: 10.3390/ijms232112981 (PMC9658848; doi:10.3390/ijms232112981)
Supplement: Supplementary file 1 [file ijms-23-12981-s001.zip › ijms-1968823-supplementary/Supplementary Table S9.pdf]

Supplementary Table S9. Expression patterns of DEGs related to flavonoid biosynthesis during adventitious root development in cucumber.

Gene id	Gene name	FPKM(the control)	FPKM(ETH)	log2FC	Gene description	Up/down
101218716	*LOC101218716*	884.4913662	497.4645709	-0.828950716	probable chalcone--flavonone isomerase 3 isoform X1	down
101207160	*LOC101207160*	463.683235	73.27810297	-2.659738715	flavonoid 3'-monooxygenase	down
101207100	*LOC101207100*	0.302406805	6.959013978	4.348192306	stemmadenine O-acetyltransferase	up
