# Supplementary material for: Identification of Key Genes during Ethylene-Induced Adventitious Root Development in Cucumber (Cucumis sativus L.)
Source: Int J Mol Sci. 2022 Oct 26;23(21):12981. doi: 10.3390/ijms232112981 (PMC9658848; doi:10.3390/ijms232112981)
Supplement: Supplementary file 1 [file ijms-23-12981-s001.zip › ijms-1968823-supplementary/Supplementary Table S1.pdf]

Supplementary Table S1. Quality statistics of transcriptome.

Sample	Raw reads	Clean reads	Clean bases	Error rate	Q20	Q30	GCpct
the control _1	45919430	45224954	6.78G	0.02	98.07	94.27	42.39
the control _2	45749502	45051114	6.76G	0.03	97.99	94.08	41.61
the control _3	47928220	47106620	7.07G	0.02	98.38	94.98	42.05
ETH_1	45413156	44594122	6.69G	0.02	98.09	94.29	42.06
ETH_2	46001162	44523656	6.68G	0.02	98.23	94.69	41.5
ETH_3	47556332	45532650	6.83G	0.02	98.19	94.62	41.2
