# Supplementary material for: Identification of Key Genes during Ethylene-Induced Adventitious Root Development in Cucumber (Cucumis sativus L.)
Source: Int J Mol Sci. 2022 Oct 26;23(21):12981. doi: 10.3390/ijms232112981 (PMC9658848; doi:10.3390/ijms232112981)
Supplement: Supplementary file 1 [file ijms-23-12981-s001.zip › ijms-1968823-supplementary/Supplementary Table S2.pdf]

Supplementary Table S2. Sequences of primers used for RT-qPCR analysis.

Gene symbol	NCBI gene accession numbers	Gene ID	Product size	Primer Sequence (5'-3')	Primers efficiency	Correlation coefficient (R^2)
CsGBE1	ACHR03000062	101215687	107	F:GACAGTGAAGGGTTGGCAGGTTG<br>R:TCATCATCTTCGGGTTTGCTCGTATC	92.15%	0.9927
Cseg9	ACHR03000028	101222401	81	F:GTTACTAGCGACGGCGAAGAAGG<br>R:ACAGCAGAGGAAAGCGAATCACTATAC	93.41%	0.9958
Cseg6	ACHR03000050	101203271	111	F:CAGTGGTGGACATAGTGGCTACAATC<br>R:GAAGACGATGCTGGAGATGGAAGTG	90.03%	0.9864
Csβ-G11-like	ACHR03000050	101214838	116	F:CTTTCGGTTTGTTCCTTTGACTGACTC<br>R:ACTTCGGATACTCACCATACACCAATG	97.39%	0.9942
CsHK2	ACHR03000083	101216058	113	F:TACCTTGGTGAAATTGCTCGTAGAGTG<br>R:TCTGGTGTGCTCAGGATGAATTGC	95.62%	0.9987
CsSS5-like	ACHR03000028	101211461	91	F:TTTACGACGAAGAATGGGCAAATGATG<br>R:GATGGAAGGCTTAACCGAGGAGTTG	91.26%	0.9092
CsPFP	ACHR03000058	101215219	83	F:TTCTCCCGCCAATCCTCCTTCTC	95.47%	0.9956

				R:GATGGATACGATGGGCTTGGACTTG		
CsPK2	ACHR03000062	101206620	81	F:CTCGTGTGGTAGACAGCATGACTAAC<br>R:CATCAAGTACAGCATTGGCAACATCAG	95.52%	0.9963
CsACS2	ACHR03000062	101209539	83	F:GATGAAAGAGGTGAGGAGCCAAGATAC<br>R:ATAACATCGCCAACGCCATAGCC	93.67%	0.9281
CsIDH	ACHR03000028	101202770	105	F:TCCTCCACCTCCTCCTCTACTCTC<br>R:AGAAGAAGAAGAAGAAGCGTAGCATCG	98.71%	0.9860
CsLACS6	ACHR03000083	101207461	82	F:TTAATTGCGAGTGTTGCTGGATGTTC<br>R:GTGCCAAAGGGAGGTACGATATGTAG	96.15%	0.9785
CsCYP450 86A1	ACHR03000058	101214516	96	F:CGCTGGACCGAGGACTTGTTTG<br>R:ACTGGAGATAGCCGATACCGAAGG	99.57%	0.9961
CsLAX5	ACHR03000006	101205750	144	F:TCTACATCATTCCTGCTCTTGCTCAC<br>R:CCAACACCCAAACCACTACAAATGC	97.79%	0.9902
CsGH3.17	ACHR03000050	101206247	84	F:TGAAAGAATTGCCAATGGTGAACCTTC<br>R:CCAGAAGTTCCTGAGCTTGTGAGAAG	98.15%	0.9969
CsSAUR50	ACHR03000083	101217643	81	F:TGTCTACGTTGGTCAACACCGAAC<br>R:TGGAGCAAGATTTGGAAAGGAGGATG	96.90%	0.9918

CsPYR1	ACHR03000083	101204882	96	F:GACGAGTTGAAGGACTTAGTAGCAGAG<br>R:TGAACACGCTGAGCAAGTAAGGAG	98.87%	0.9985
CsCS-ERS	ACHR03000006	101205786	131	F:AAGTTGCTTGTGCTATTGTATCGTGTG<br>R:GCCCATCTCCCTGTCAAGTTGTTC	103.11%	0.9891
CsERFC3	ACHR03000014	101206564	129	F:CGGAGATACGAGACTCAACCAGAAATG<br>R:AATTAAGAACGGCGGCGGAACC	98.03%	0.9936
CsBAK1	ACHR03000006	101216386	132	F:GGGTCGTTTGGCTGATGGTTCTC<br>R:AGACGGAGTAGATTACGGTGGACAG	95.48%	0.9915
CsPAL	ACHR0300006	101218856	148	F:AGCATCATCCTGGACAGATTGAAGC<br>R:TGCGGTGAAGTTCTAAGAGCGTAAC	99.24%	0.9979
CsPAO	ACHR03000028	101213059	136	F:CCTTCTAGTCTCTCGTTTGTGGTTTCC<br>R:AGTGAGTGGATCTAGTGGATGGTGAG	101.05%	0.9966
CsAt4g34880	ACHR03000006	101204792	136	F:GCTGACAGAGAACGAGAGGCTAATAAG<br>R:TAGACCCAAGCAATGCGAATGATCC	92.08%	0.9816
Csnad5	HQ860792	11123919	148	F:CGCACAGATAGGATCGCATACTTGG<br>R:AGCCGTAGGTGGGTATTCAAATAAAGG	99.45%	0.9906
CsF3'M	ACHR03000058	101207160	103	F:ATCGTGGAGGAGCATCGGAATTTG	102.28%	0.9822

				R:TCTCATCTTCACCGTCATCCTTCATTG		
CsHCT	ACHR03000014	101207100	135	F:ACCCTGAGTTTCTGAGACAATTCCAAG<br>R:CGTCATAAAGAGGAAGTCGGCAGAG	89.07%	0.9928
